# Diagnostic journey of chronic eosinophilic pneumonia masked as lung cancer: a rare case report

**DOI:** 10.1097/MS9.0000000000001296

**Published:** 2023-09-22

**Authors:** Shailendra Katwal, Sarita Lamsal, Sushmita Bhandari, Shital Khanal

**Affiliations:** a Department of Radiology, Dadeldhura Subregional Hospital, Dadeldhura; b Nepalgunj Medical College, Nepalgunj; c Shankar Nagar Health Post, Butwol; d Institute of Medicine, Kathmandu, Nepal

**Keywords:** case report, chronic eosinophilic pneumonia, eosinophilic microabscess, lung malignancy, prednisolone

## Abstract

**Introduction and Importance:**

Chronic eosinophilic pneumonia (CEP) is an idiopathic condition characterized by unusually high eosinophil infiltration in the lungs’ interstitium and alveolar spaces. It is extremely rare, accounting for fewer than 3% of all interstitial lung diseases. CEP is frequently misdiagnosed as lung cancer, which can have catastrophic consequences for sufferers. When assessing patients with lung disease, doctors should be aware of CEP’s symptoms and take its prognosis into account because it is a curable disorder.

**Case Presentation:**

A 40-year-old female presented in the outpatient department of gynecology with a history of abnormal vaginal bleeding for 3 months and mild shortness of breath without any other significant medical history or being under any medications. Physical examination findings were not significant.

**Clinical Discussion:**

Ultrasound revealed adenomyosis and a hysterectomy was planned. Chest radiograph revealed lung mass and computed tomography scan showed a well-defined mass with a pleural-based nodule. Histopathology revealed interstitial fibrosis and eosinophilic microabscesses. CEP was diagnosed and oral prednisolone was started with a 0.5 mg/kg/day dose. Chest radiographic abnormalities resolved after one month of treatment. Currently, she is asymptomatic.

**Conclusion:**

Early recognition and diagnosis of lung masses are essential for prompt treatment with corticosteroids. CEP can mimic lung malignancy and should be considered in patients with related symptoms.

## Introduction

Highlights

- Chronic eosinophilic pneumonia (CEP) is a rare but significant interstitial lung disease frequently misdiagnosed as lung cancer.
- In our case, the diagnostic journey began with a suspicion of lung cancer, but histopathology revealed CEP, which was treated successfully with oral prednisolone.
- Healthcare providers should consider CEP in patients with lung masses and related symptoms to ensure timely and appropriate management.

Chronic eosinophilic pneumonia (CEP) is an idiopathic disorder characterized by an abnormally high level of eosinophil infiltration in the interstitium and alveolar spaces of the lungs^1^. It is an uncommon condition, accounting for less than 3% of interstitial lung diseases^2^. It is more common in females, with a male-to-female ratio of 1:2^2^.

After maturation through cytokines like IL-3 and IL-5, eosinophils play a role in both innate and adaptive immunity. Eosinophilic disorders primarily result from toxins and chemokines. Their migration to the lungs is mainly influenced by IL-5 and eotaxin. Recent research also indicates the involvement of IL-25 in chronic lung inflammation in cases of CEP. Elevated levels of osteopontin in bronchoalveolar lavage and the presence of eosinophilic granules in histopathological findings also suggest their role in inflammation^2^.

Carrington *et al*.^3^ first described CEP in a group of females who presented with respiratory symptoms, lung imaging showing opacities, and prominent eosinophils observed in biopsy specimens.

The incidence of lung masses is increasing, and although most of them are tumors, either benign or malignant, not all of them are solid masses. Various conditions, including lung cancer, hamartomas, lung abscesses, granulomas, and eosinophilic pneumonia, among others, can present as nodules in the lungs^4^. CEP should be considered in a patient with nonspecific subacute to chronic respiratory symptoms, peripheral eosinophilia, and peripheral parenchymal opacities in the upper lobes^5,6^. In this article, we describe a case of CEP whose imaging findings revealed a lung mass that mimicked lung malignancy. To the best of our knowledge, there is only one case report with a presentation similar to this one^4^.

## Case presentation

**Figure 1:**
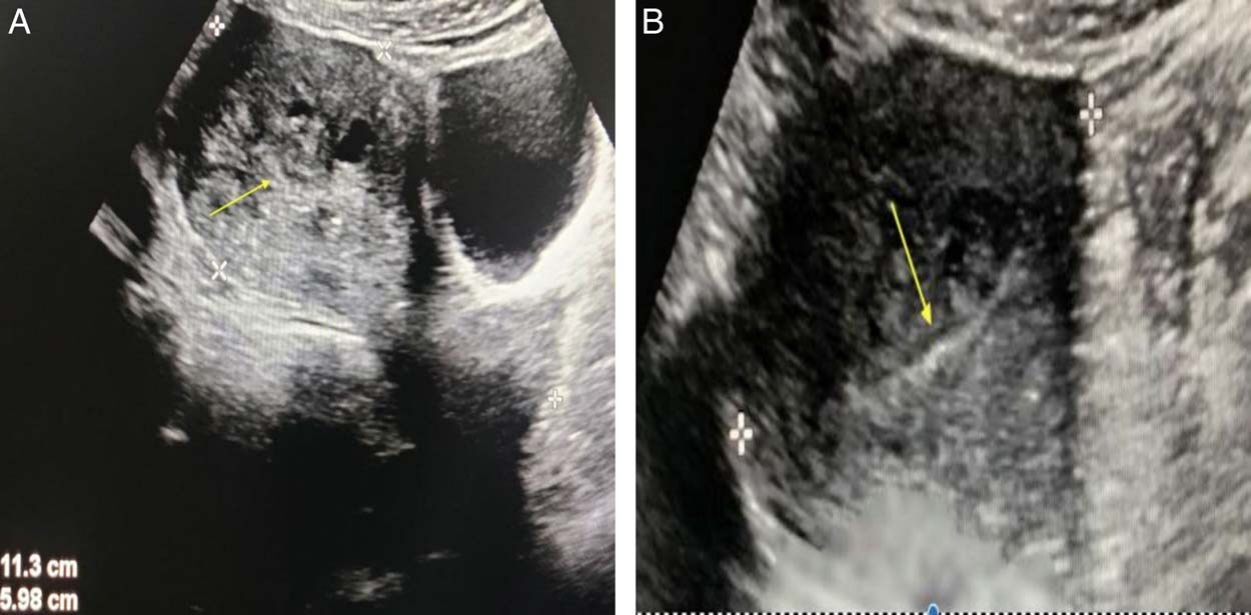
Longitudinal (A) and transverse (B) gray scale ultrasound image of the uterus showing asymmetrically thickened anterior myometrial wall with multiple anechoic areas in the myometrium. Yellow arrow pointing to the endometrium.

**Figure 2:**
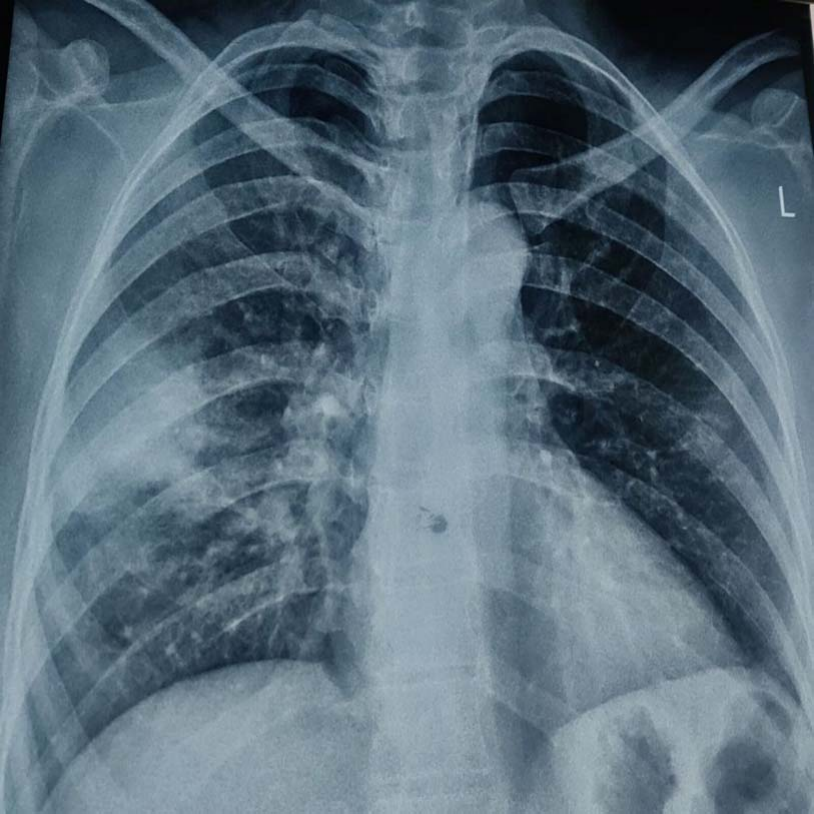
Chest X-ray posteroanterior view showing well-defined radiopaque mass with circumscribed medial margin in the peripheral aspect of the right middle zone.

**Figure 3:**
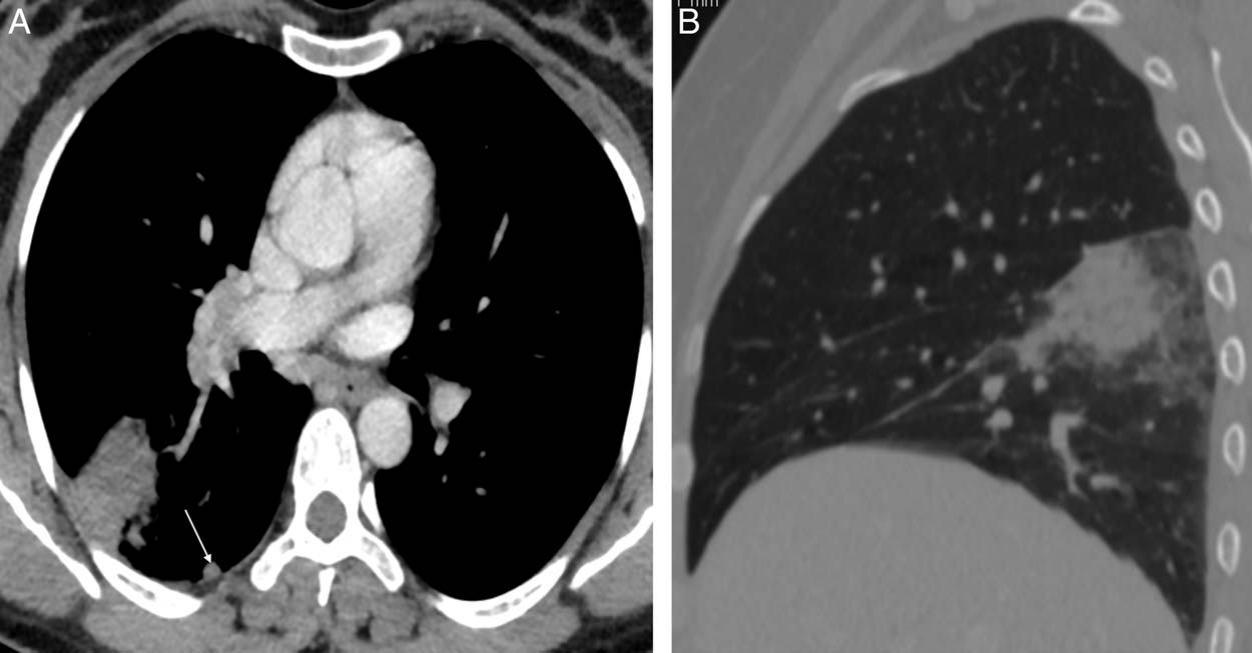
(A) Relatively well-defined mild enhancing soft tissue density mass in the peripheral aspect of the right lower lobe with enhancing pleural-based subcentimetric nodule adjacent to it (white arrow). (B) The sagittal lung window of the right lung shows soft tissue density mass in the superior aspect of the right lower lobe with adjacent ground-glass opacities and septal thickening, giving a crazy pavement pattern.

A 40-year-old female presented at the gynecology outpatient department with a history of abnormal vaginal bleeding for 3 months, accompanied by crampy abdominal pain. She had no significant medical history, was not on any medications, and did not report fever, cough, chest pain, weight loss, or travel to tropical areas. There was no family history of tuberculosis, and she lived in a well-ventilated house. Physical examination findings were not significant. Pelvic examination revealed a normal vagina, vulva, and normal-sized uterus. Ultrasound of the abdomen and pelvis was done, which revealed asymmetric myometrial wall thickness with multiple anechoic areas in the myometrium features suggestive of adenomyosis (Fig. 1A, B) after which a hysterectomy was planned. During the preoperative evaluation, the patient complained of occasional shortness of breath, following which a chest X-ray was done. Chest X-ray revealed a well-defined round-to-oval lung mass in the peripheral aspect of the right middle zone with circumscribed medial margin and obscured lateral margin (Fig. 2). Considering its high prevalence in developing countries, tuberculosis was also considered as a potential differential diagnosis. However, the patient did not exhibit symptoms such as fever, cough, weight loss, or any history of contact with tuberculosis. Additionally, the erythrocyte sedimentation rate (ESR) was slightly elevated, around 30. Subsequently, a contrast-enhanced computed tomography (CECT) of the chest was done. Computed tomography showed mildly enhancing soft tissue density mass in the right lower lobe with a surrounding crazy pavement pattern, pleural-based enhancing nodule adjacent to the mass, and bilateral hilar lymphadenopathy (Fig. 3A, B). With the suspicion of lung malignancy, a lung biopsy was done under ultrasound guidance using the Tru-Cut needle. Histopathology report showed the section of lung parenchymal tissue with interstitial fibrosis and dense interstitial infiltrate of lymphocytes and eosinophils, eosinophilic microabscesses, eosinophilic aggregates in alveoli, and fibroconnective tissue plugs. Features of vasculitis were not seen. Eosinophilia and mild anemia were detected during the laboratory workup, and subsequently, the immunoglobulin E (IgE) level was assessed and found to be elevated (Table 1). With these features, parasitic infestation and CEP was considered differential diagnosis. The parasitic infestation was ruled out with stool studies and the absence of travel history. Based on these features, CEP was diagnosed. Subsequently, oral prednisolone was started with a 0.5 mg/kg/day dose. Subjective and radiographic improvement was seen after 48 h of treatment. Chest radiographic abnormalities resolved after one month of treatment, following which the prednisolone dose was decreased to 0.25 mg/kg/day, continued for another 8 weeks, and then tapered. No adverse event was noted during the course of treatment. Currently, she is asymptomatic.

**Table 1:** Laboratory result of the patient.

Test	Results	Normal values
Total leukocyte count	6000	4000–11 000/mm^3^
Neutrophil	60	40–70%
Lymphocyte	24	20–45%
Eosinophil	30	1–6%
Hemoglobin	12	13–18 g/dl
MCV	89	80–100 fl
MCH	31	26–34 pg
RBC count	4.4	4.5–5.5 million/mm^3^
Platelet count	1.8	1.5–4.5×10^5^/mm^3^
IgE	1200 IU/ml	<150 IU/ml
ESR	30	0–20 mm/h in female
CRP	6	<0.3 mg/dl
Peripheral blood smear	Increased eosinophil count with no blast cells or any dysplastic cells	

CRP, C-reactive protein; ESR, erythrocyte sedimentation rate; IgE, immunoglobulin E; MCH, mean corpuscular hemoglobin; MCV, mean corpuscular volume; RBC, red blood cell.

## Discussion

CEP falls under the class of eosinophilic lung disease. Eosinophilic lung disease can be classified into known causes (medications, allergic bronchopulmonary aspergillosis, and parasitic infestation), unknown causes (acute eosinophilic pneumonia, CEP, and Loeffler syndrome), and eosinophilic vasculitis (eosinophilic granulomatosis with polyangiitis)^7^. The diagnosis of CEP requires pulmonary eosinophilia in bronchoalveolar lavage or lung biopsy and exclusion of other causes of eosinophilic lung diseases^7^.

CEP presents with nonspecific respiratory symptoms. Clinical features include a history of atopy, gradual onset of productive cough, fever, shortness of breath, weight loss, night sweats, wheezing, and crackles. Nearly 50% of patients with CEP have a history of asthma^7^. The only symptom in our case was mild, occasional shortness of breath for 2 months and she had no history of atopy.

Lab test shows peripheral blood eosinophilia in 90% of cases and elevated immunoglobulin E levels in 50% of cases^5,8^. There can be only mild elevation of peripheral blood eosinophils in CEP, so if radiological features are consistent, diagnosis of CEP should be considered^9^. In our case, the eosinophil differential count was elevated and was 30%.

Common imaging finding involves ground-glass and consolidative opacities, typically in peripheral and upper lung fields. Less common radiologic findings include lymphadenopathy and pleural effusion. After the disease improves, imaging may reveal vertical bands parallel to the pleural surface and pulmonary fibrosis^5^. The radiologic finding was unusual in our case. CECT chest showed well-defined enhancing soft tissue density lung mass with pleural-based enhancing nodule, which was thought to be due to malignancy. Similar to our case, a case report written by Amoda *et al.* had supra and infra hilar mass with peripheral consolidation with suprahilar, hilar, infra hilar, and mediastinal lymphadenopathy with unilateral pleural effusion^4^.

In our case, although the eosinophil count was very high, both eosinophilic vasculitis and allergic bronchopulmonary aspergillosis were ruled out. The patient did not exhibit constitutional features like weight loss, fatigue, or fever, and there were no signs of paranasal sinus involvement or peripheral neuropathy. The biopsy findings did not support the presence of vasculitis. Furthermore, the absence of bronchiectasis changes in the lung parenchyma and the presence of mild shortness of breath rather than typical asthmatic symptoms led to the ruling out of allergic bronchopulmonary aspergillosis. Additionally, there was no travel history, and a normal stool examination ruled out the parasitic infestation.

## Conclusion

The differential diagnoses in a patient presenting with a lung mass can be narrowed according to their history and presenting illness. CEP can present as a lung mass mimicking lung malignancy and should be considered in patients with related symptoms, peripheral eosinophilia, and a lung mass. Early recognition and diagnosis is essential, and prompt treatment with corticosteroid is the mainstay of therapy.

## Ethical approval

This case report did not require review by the ethical committee.

## Consent

Written informed consent was obtained from the patient for the publication of this case report and the accompanying images. A copy of the written consent is available for review by the Editor-in-Chief of this journal on request.

## Sources of funding

None.

## Author contribution

S.K.: conceptualization, as mentor and reviewer for this case report and data interpretation; S.L.: contributed to performing literature review and editing; S.B.: contributed to writing the paper and reviewer for this case; S.K.: contributed to writing the paper. All authors have read and approved the manuscript.

## Conflicts of interest disclosure

There are no conflicts of interest.

## Research registration unique identifying number (UIN)

The case report at hand is not a first-in-man case report of a novel technology or surgical technique; therefore, registration of these case reports, according to the Declaration of Helsinki 2013, is not required.

## Guarantor

Shailendra Katwal.

## Provenance and peer review

Not commissioned, externally peer-reviewed.
